# Influence of PMSG on Superstimulation and Embryo Development Following Somatic Cell Nuclear Transfer in Holstein Cows in the United Arab Emirates

**DOI:** 10.3389/fvets.2022.895325

**Published:** 2022-04-26

**Authors:** Young-Bum Son, Yeon Ik Jeong, Mohammad Shamim Hossein, Xianfeng Yu, Per Olof Olsson, Mina Kang, Huijeong Kim, Yura Bae, Alex Tinson, Kuhad Kuldip Singh, Singh Rajesh, Al Shamsi Noura, Woo Suk Hwang

**Affiliations:** ^1^UAE Biotech Research Center, Abu Dhabi, United Arab Emirates; ^2^Abu Dhabi Biotech Research Foundation, Seoul, South Korea; ^3^Jilin Provincial Key Laboratory of Animal Model, Jilin University, Changchun, China; ^4^Hilli E.T. Cloning and Surgical Centre Presidential Camels and Camel Racing Affairs, Abu Dhabi, United Arab Emirates; ^5^Department of Biology, North-Eastern Federal University, Yakutsk, Russia

**Keywords:** Holstein cows, PMSG, superstimulation, ovum pick-up, somatic cell nuclear transfer

## Abstract

The present study investigated the effect of superstimulation to improve *in vitro* embryo production in the Gulf area, where the temperature is high. Holstein cows were classified into the control and superstimulation groups. Superstimulation was induced with a single intramuscular injection of pregnant mare serum gonadotropin (PMSG; 2500 IU) on day 14 of the estrus cycle (day 0; estrus). The development of follicles was evaluated by ultrasonography of the ovaries daily. At 40 h after the PMSG injection, oocytes were collected by the ovum pick-up (OPU) technique. OPU was performed at the same stage of the estrus cycle in the control group as in the superstimulation group. The number of follicles with a diameter of more than 6 mm and the number of retrieved cumulus-oocyte complexes were significantly higher in the superstimulation group than in the control group. Furthermore, the maturation rate was higher in the superstimulation group than in the control group. Cloned embryos were produced by somatic cell nuclear transfer using matured oocytes. The cleavage and blastocyst formation rates were significantly higher in the superstimulation group than in the control group. In conclusion, a single injection of PMSG can facilitate the efficient production of cloned cow embryos.

## Introduction

The production of animals with superior genetic value is important for the success of the livestock industry according to the economic characteristics of the breed. Somatic cell nuclear transfer (SCNT), a technique for preserving superior traits in cattle, has been studied as an important alternative to enhance reproductive efficiency (1). Various factors affect the efficiency of SCNT, including donor cell type and cycle, and oocyte activation (2). Among them, the quantity and quality of oocytes are crucial factors for improving cloning (2). Holstein cows are monotocous animals with a long gestation period and produce small numbers of calves throughout their lives. Numerous primordial follicles exist in the ovary and have the potential to produce offspring, but only a small number of oocytes that mature and ovulate give rise to calves. The potential reproductive capacity of females can be improved by artificially maturing and recovering oocytes from females with excellent genetic traits and using these oocytes to produce live offspring.

The appropriate environmental temperature for Holstein cows is 5–25°C (3). Stress occurs when the environmental temperature exceeds the appropriate temperature range, which affects the health and reproduction of Holstein cows. Furthermore, under heat stress, fertility is reduced due to hormonal changes (4). Heat stress caused by high environmental temperatures also adversely affects the efficiency of follicle generation and follicle quality (5). Several studies reported that heat stress during the estrus cycle, in which small follicles grow into pre-ovulatory follicles, decreases follicular growth (5, 6). In the Arabian Peninsula, Holstein cows have only recently been raised and milked, but reproduction is difficult due to the unique climate characteristics of this region. Therefore, the present study determined whether cloning after superstimulation would to improve reproductive efficiency in heat-stressed cows in the high temperature environment of the United Arab Emirates (UAE).

In general, pregnant mare serum gonadotropin (PMSG) and follicle-stimulating hormone (FSH) are used for superstimulation of Holstein cows. FSH must be administered twice per day for 4–5 days for hyperstimulation due to its short half-life (7, 8). This is stressful for cows because it requires frequent injections, which is also inconvenient. Additionally, FSH stimulation requires the administration of FSH every 12 h. It is consequently associated with increased amounts of labor and costs. PMSG is a complex glycoprotein containing sialic acid whose administration stimulates secondary follicles to improve the development of follicular oocytes. Furthermore, the half-life of PMSG is longer than that of FSH and thus a single intramuscular (IM) injection of PMSG can be effective (9, 10). This study was conducted to investigate the superstimulation efficiency and embryo developmental capacity of oocytes through SCNT after a single PMSG intramuscular injection in the United Arab Emirates.

## Materials and Methods

### Chemicals

All chemicals used were purchased from Sigma (St. Louis, MO, USA) unless otherwise noted.

### Animals

This study was conducted between April 2021 and June 2021. All experiments were performed in a commercial dairy farm (Al ain dairy farm) in the UAE. During this period, the average minimum temperature was 26.32°C and the average maximum temperature was 40.90°C (https://weather.com). The control group and the superstimulation group contained 229 and 133 Holstein cows, respectively. Female Holstein cows aged between 2 and 3 years and weighing 350 kg were used. Oocytes were collected simultaneously between 10 am and 2 pm from both the control and superstimulation groups. Animal experiments were conducted according to the animal study guidelines, which were approved by the ethics committee of the UAE Biotech Research Center (Approval No.: UAEBRC-B01). These guidelines comply with the ARRIVE guidelines, the UK Animals (Scientific Procedure) Act, 1986, and EU Directive 2010/63/EU.

### Superstimulation, OPU, and *in vitro* Maturation

**Figure 1:**
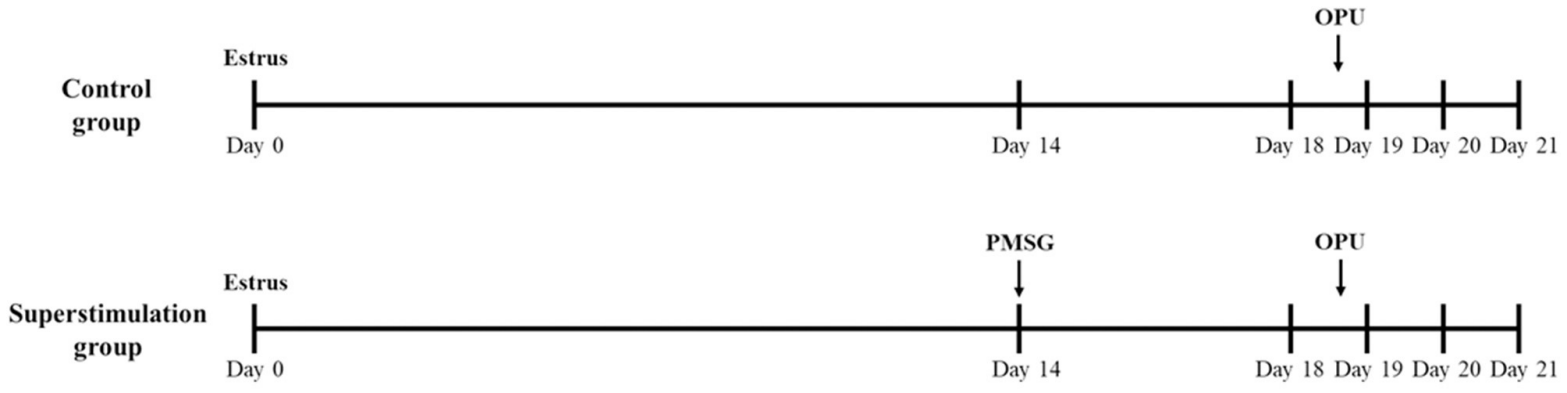
Superstimulation protocol to investigate the effect of PMSG injection prior to ovum pick-up (OPU) in Holstein cows. PMSG, pregnant mare serum gonadotropin; OPU, ovum pick-up.

On day 14 of the natural estrus cycle (estrus; day 0), oocyte donors were injected with 2500 IU PMSG (Ceva, Libourne, France) to stimulate the ovaries (Figure 1). The oocyte donors were sedated with 10 mg of xylazine (Ceva, Libourne, France). Cumulus-oocyte complexes (COCs) were obtained using an Aloka Ultrasound Unit (Aloka, Tokyo, Japan) and a needle guide (Aloka) from follicles (≥6 mm) at 40 h after PMSG injection. The control group did not receive any hormonal treatment, and COCs were collected at the same stage of the estrus cycle as in the superstimulation group (Figure 1). Lumen needle (60 cm, 18-gauge) were used for follicle oocyte retrieval, along containing 15 ml capped tubes with 2 ml OPU solution (IVF Bioscience, Falmouth, UK) using a regulated vacuum pump. The *in vitro* maturation of oocytes was conducted according to the instruction of the manufacturer of IVM media. For IVM, COCs were cultured at 38°C with 5% CO_2_ in a humidified atmosphere for 22 h with IVM media (IVF Bioscience, Falmouth, UK).

### Oocyte Nuclear Maturation Evaluation and Somatic Cell Nuclear Transfer

Donor cells were prepared using a conventional primary cell culture system as described previously (11). Evaluation of the maturation stage of the collected oocytes and SCNT were conducted as described previously with minor modifications (12). Cumulus cells were removed from oocytes with 0.1 % hyaluronidase. After denuding, metaphase II phase oocytes were stained with 5 μg/ml bisbenzimide for 3 min. The stained oocytes were enucleated by squeezing out the first polar body and the metaphase II spindle plate together with some of the surrounding cytoplasm using a glass pipette. A trypsinized fibroblast with a smooth cell surface was transferred into the perivitelline space of an enucleated oocyte. The couplets were equilibrated in 0.26 M mannitol, 0.1 mM MgSO_4_, 0.5 mM HEPES, and 0.05% (w/v) bovine serum albumin with two direct current pulses of 1.8 kV/cm for 15 μsec using a BTX Electro Cell Manipulator (BTX Inc., San Diego, CA, USA). After fusion, the reconstructed embryos were treated with 5 μM ionomycin for 4 min and with 2.0 mM 6-dimethylaminopurine in BO-IVC (IVF Bioscience) in a humidified incubator containing 5% CO_2_ at 38°C for 4 h. Following activation, 6–8 embryos were cultured in an oil-covered droplet at 38°C in a humidified atmosphere containing 5% CO_2_ and 5% O_2_.

### Statistical Analysis

Between-group comparisons were performed using the independent T-test of variance with SPSS for Windows (version 23; SPSS Inc., Chicago, Il, USA). Statistical significance between mean values was assessed using Duncan's multiple range test. *P*-values < 0.05 were considered statistically significant. Data are presented as the mean ± standard error (SE).

## Results

Data concerning follicular growth and collected oocytes from 362 donors in the superstimulation and control groups are presented in Table 1. The average number of follicles (≥6 mm) and collected oocytes were significantly higher in the superstimulation group than in the control group (Table 1). However, the recovery rate was similar in between the superstimulation and the control groups (Table 1). The maturation rate of oocytes was significantly higher in the superstimulation group than in the control group (Table 1). The percentage of abnormal oocytes did not differ significantly between the control and superstimulation groups. The results of embryo development are presented in Table 2. We confirmed that the fusion rates of SCNT embryos were similar between the superstimulation group and the control group (Table 2). Additionally, the cleavage and blastocyst formation rates following SCNT using these oocytes were significantly higher in the superstimulation group than in the control group (Table 2).

**Table 1:** Effects of superstimulation on oocytes after ovum pick-up in Holstein cows.

**Group**	**No. of**	**No. of**	**No. of**	**Recovery**	**No. of oocytes (%)**		
	**donors**	**≥6 mm follicles**	**collected oocytes**	rate (%)*
					**MII phase^†^**	**Immature^**‡**^**	**Abnormal**
Control	229	731 (3.18 ± 0.18)^a^	543 (2.24 ± 0.18)^a^	70.82 ± 3.12	387 (72.62 ± 1.91)^a^	146 (25.82 ± 1.79)^a^	10 (1.55 ± 0.32)
Superstimulation	133	764 (4.73 ± 0.45)^b^	587 (4.40 ± 0.30)^b^	74.77 ± 2.53	476 (80.81 ± 0.91)^b^	98 (17.12 ± 1.04)^b^	13 (2.07 ± 0.38)

*Different superscript letters (a and b) represent significance (P < 0.05)*.

* *No. of collected oocytes/no. of follicles*.

*MII: metaphase II oocytes*.

‡ *Immature: metaphase I, germinal vesicle, and germinal vesicle breakdown oocytes*.

**Table 2:** Effects of superstimulation on the development potential of embryos generated by somatic cell nuclear transfer in Holstein cows.

**Group**	**Nuclear transfer**			
	**No. of oocytes**			
	**Reconstructed oocytes**	Fused oocytes (%)*	**Cleaved oocytes (%)^†^**	**Blastocysts (%)^**‡**^**
Control	387	271 (69.99 ± 1.10)	193 (70.90 ± 0.60)^a^	53 (18.06 ± 1.54)^a^
Superstimulation	476	357 (73.78 ± 2.35)	261 (73.71 ± 0.80)^b^	78 (23.82 ± 2.17)^b^

*Different superscript letters (a and b) represent significance (P < 0.05)*.

* *The fusion rate was calculated as the percentage of reconstructed oocytes that underwent fusion*.

†,‡ *The cleavage and blastocyst formation rates were calculated as the percentages of fused oocytes that underwent cleavage and developed into blastocysts*.

## Discussion

Superstimulation of oocyte donors is the first and crucial step in any *in vitro* embryo production system. Various hormones with stimulatory effects on the ovary, such as FSH and PMSG, are used in standard ovarian stimulation protocols for Holstein cows. However, the reproduction capacity of cloned embryos derived from oocytes collected from superstimulated cows is not evaluated in the UAE, which has a very hot climate. It is well established that high temperature has a detrimental effect on oogenesis and the quality of oocytes. Therefore, this study investigated the *in vitro* maturation and developmental capacity of embryos derived from oocytes obtained from PMSG-superstimulated donors cows using SCNT following OPU. Our results revealed that PMSG treatment of Holstein cows resulted in oocytes that developed into cloned blastocyst under the unique environmental conditions of the United Arab Emirates (UAE).

Environmental temperature is one of the most crucial factors according cattle reproduction (13). Several studies have reported that heat stress negatively effects reproduction in Holstein cows (14–16). Heat stress affects estrus duration, uterine function, and endocrine status (14–16). Furthermore, prolonged heat stress can affect early embryonic development and survival (17, 18). Heat stress during the estrus cycle of Holstein cows reduces the activity of granulosa cells and hinders follicular development (7). Furthermore, it has been reported that heat-stressed Holstein cows have a decreased follicle diameter and reduced fertility (19), and that heat stress during the early estrus cycle results in reduced follicular growth (5). The appropriate environmental temperature for Holstein cow is 5 to 15°C, and the temperature at which cows can maintain fertility without additional physiological changes is 5 to 25°C (3, 20) after they have been adapted to a high temperature environment. Furthermore, cows are subject to severe heat stress when the daily minimum temperature does not fall below 20°C (21). The present study demonstrated that the numbers of follicles (≥6 mm), collected oocytes, and *in vitro* maturation capacity increased following PMSG treatment. However, the number of follicles (≥6 mm), oocytes were lower in both the control and superstimulation groups than in a previous study, and this difference was thought to be due to heat stress (22–26). As described above, the minimum temperature during the period of this study was over 20°C, and the maximum temperature approached 40°C.

Oocyte developmental capacity gradually increases during follicular growth, and the capacity to develop to the blastocyst stage correlates with gene expression profiles during follicular growth (27, 28). Normal ovaries are strong enough to withstand superstimulation for the development of many follicles rather than a single follicle during the estrus cycle. Furthermore, ovary superstimulation increases the quality of the collected oocytes, thereby increasing the *in vitro* embryonic developmental capacity (27). Several studies using genetic profiling have revealed that oocytes collected from superstimulated ovaries differed from those from non-stimulated ovaries in mouse and cows (29, 30). *In vitro* blastocyst formation is also more efficient in superstimulated cows than in non-stimulated cows (29, 30). This study demonstrated that oocytes collected from superstimulated ovaries showed enhanced *in vitro* maturation and blastocyst formation capacity (29, 30). Therefore, we hypothesize that superstimulation using PMSG before OPU affects the developmental potential of oocytes. The results of our study are consistent with those of previous studies (7). Oocytes in the superstimulation group exhibited enhanced IVM and a better potential to develop to the cleavage and blastocyst stages following SCNT than oocytes in the control group (Tables 1, 2).

Although further research will be necessary to determine the effect of superstimulation on heat stress, our results showed that, follicle maturation, oocyte *in vitro* maturation, and embryonic development following somatic nuclear transfer were more efficient after superstimulation of Holstein cows in the UAE. In conclusion, the present study demonstrated that a single IM injection of PMSG, which is less stressful for Holstein cows, cheaper, and less labor intensive than injection of other hormones, is a useful method for superstimulation of Holstein cows in the UAE.

## Data Availability Statement

The original contributions presented in the study are included in the article/supplementary material, further inquiries can be directed to the corresponding author.

## Ethics Statement

The animal study was reviewed and approved by the Ethics Committee of the UAE Biotech Research Center (Approval No.: UAEBRC-B01). Written informed consent was obtained from the owners for the participation of their animals in this study.

## Author Contributions

Y-BS and WH: conceptualization. Y-BS, YJ, XY, PO, MK, HK, YB, AT, KS, SR, AN, and WH: methodology. Y-BS, YJ, MH, MK, and AT: investigation. Y-BS, PO, and AN: validation. Y-BS and KS: formal analysis. YJ, HK, YB, AT, KS, and SR: resources. Y-BS: writing—original draft preparation. Y-BS, MH, PO, and WH: writing—review and editing. WH: funding acquisition. All authors have read and agreed to the published version of the manuscript.

## Conflict of Interest

The authors declare that the research was conducted in the absence of any commercial or financial relationships that could be construed as a potential conflict of interest.

## Publisher's Note

All claims expressed in this article are solely those of the authors and do not necessarily represent those of their affiliated organizations, or those of the publisher, the editors and the reviewers. Any product that may be evaluated in this article, or claim that may be made by its manufacturer, is not guaranteed or endorsed by the publisher.
